# Exploring the Limits of Passive Macromolecular Translocation through Phospholipid Membranes

**DOI:** 10.1021/acs.biomac.5c01234

**Published:** 2025-09-12

**Authors:** Ekaterina Kostyurina, Ralf Biehl, Margarita Kruteva, Alexandros Koutsioubas, Henrich Frielinghaus, Nageshwar Rao Yepuri, Stephan Förster, Jürgen Allgaier

**Affiliations:** † Jülich Centre for Neutron Science (JCNS-1), 28334 Forschungszentrum Jülich GmbH, Leo Brandt Straße, Jülich 52425, Germany; ‡ Jülich Centre for Neutron Science (JCNS), Heinz Maier-Leibnitz Zentrum (MLZ), Lichtenbergstrasse 1, Garching 85748, Germany; § National Deuteration Facility, 5419 Australian Nuclear Science and Technology Organisation, New Illawarra Rd, Lucas Heights, New South Wales 2234, Australia

## Abstract

Transportation of active macromolecules through cell membranes is an essential biological process. However, for hydrophilic macromolecules, the hydrophobic interior of lipid bilayers suppresses the passive translocation, and there are only few cases reported. We use alternating amphiphilic polymers (AAPs) in which the sizes of the hydrophilic and hydrophobic units can be varied over a broad range, keeping the polymers water-soluble. For small units, the macromolecules show a homopolymer-like character. Pulse field gradient NMR and neutron reflectivity measurements show that the chains have a high solubility in the membrane hydrophobic interior that allows the chains to passively translocate. Increasing the length of the hydrophilic units leads to more polar AAPs with low membrane solubility and a reduced translocation speed. If hydrophilic and hydrophobic moieties are increased in size, the AAPs have a strong amphiphilic character and adsorb to lipid membranes only with their hydrophobic units, have a high membrane concentration, and have an overall fast translocation kinetics.

## Graphical abstract

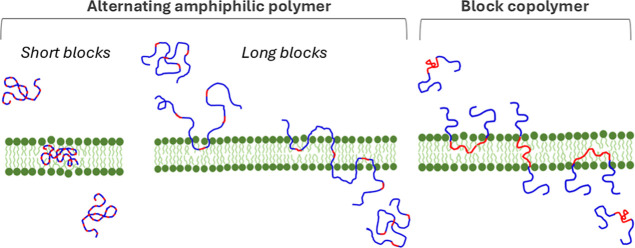

## Introduction

The transport of molecules across cell membranes is a critical process in biology. However, the cell membrane has a barrier function, where the hydrophobic interior of the lipid bilayer prevents passive cross-membrane diffusion of polar molecules. Therefore, mostly small molecules of moderate polarity can cross lipid membranes by passive diffusion,^1,2^ whereas macromolecules translocate through the membrane mostly via endocytosis and exocytosis pathways or,^3^ in the case of highly charged molecules, via pore formation.^4^ Both of these mechanisms are not ideal when the task is to deliver molecules to the cell interior because endocytosis pathways require a second delivery step—endosomal escape—and the pore formation pathway is harmful to the cells due to the formation of leaks in the membranes. A very limited number of macromolecules are known to cross the cell membrane by passive diffusion. Among them are cell-penetrating peptides, which enable translocation via conformational changes of the secondary structure, resulting in a hydrophobic peptide surface inside the membrane.^5^ Translocation of alternating and random polymers was predicted by theory^6^ and also observed for a few synthetic amphiphilic polymers.^7–9^ However, most amphiphilic polymers, especially di- and triblock copolymers, decorate lipid membranes via the insertion of the hydrophobic blocks into the membrane interior.^10,11^ So far, only in one case was translocation reported for PEG-PPG-PEG triblocks (Pluronic) at elevated temperatures.^12^ On the other hand, hydrophilic nonionic polymers show no translocation due to insolubility in the membrane interior. Based on these observations, a successful candidate for translocation must be soluble in both the aqueous environment and the membrane interior and have relatively short hydrophobic units to avoid trapping in the membrane interior. In a previous study, we showed that nonionic alternating amphiphilic polymers (AAPs) of intermediate polarity can cross lipid membranes by passive diffusion in the time scale from minutes to hours depending on the AAP length, lipid composition, temperature, and other factors. In that study, we used AAPs consisting of hydrophobic dicarboxylic acids and hydrophilic polyethylene glycol (PEG) oligomers. A unique advantage of such AAPs is the ability to tune their polarity continuously and over a very broad range by varying the hydrophobic/hydrophilic ratio.^13^ The AAPs show lower critical solution temperature (LCST) behavior. The transition temperature is a good measure for the average polymer polarity; the lower the LCST is, the more hydrophobic the polymer is. We showed that the AAPs having an LCST in the range of 25–40 °C and relatively short hydrophilic and hydrophobic units are well suited for the translocation application.^9^ These findings are in qualitative agreement with simulation studies on the translocation of amphiphilic homopolymers and amphiphilic random copolymers.^14,15^ In medical applications, there is a need for the compounds to work at body temperature, so the AAPs with LCST values above 40 °C are needed. This is especially the case if hydrophobic pharmaceutically active compounds are connected to an AAP.^9^ In order to keep the adduct water-soluble and to prevent the membrane interior from acting as a hydrophobic trap, more hydrophilic but still translocating polymers are needed.

In this work, we explored the translocation phenomenon for more hydrophilic AAPs. AAPs with a given LCST can have very different polarity profiles along the chain, which are expected to strongly influence the translocation behavior. Increasing the sizes of the hydrophilic and hydrophobic units leads to the limiting case of long, blocky structures like di- or triblock copolymers, where translocation is expected to be hindered due to the incompatibility of the hydrophilic blocks with the membrane interior.^11^ However, in one study, translocation of Pluronic polymers has been observed.^12^ Therefore, in this study, Pluronic-F127 is included as a limiting case. The effect of AAP overall polarity is systematically studied using AAPs with short units and, consequently, an almost homogeneous polarity profile to minimize additional dependence on the AAP unit length.

## Materials and Methods

### Materials

1-Palmitoyl-2-oleoyl-*sn*-glycero-3-phosphocholine (POPC) was purchased from Avanti Polar Lipids and used without further purification. 1-Palmitoyl-*d*_31_-2-oleoyl-*d*_33_-*sn*-glycero-*d*_5_-3-phosphocholine-*d*_13_ (POPC-*d*_82_) was purchased from the ANSTO national deuteration facility. The synthetic procedures for producing them are published.^16^ Pluronic-F127 was purchased from Sigma-Aldrich and used without further purification. The AAPs listed in Scheme 1 were synthesized as described in the previous publication.^13^ Synthesis and fractionation of P­(C_4_EG_6_) and P­(C_4_EG_9_) are described in Supporting Information.

**Scheme 1:**
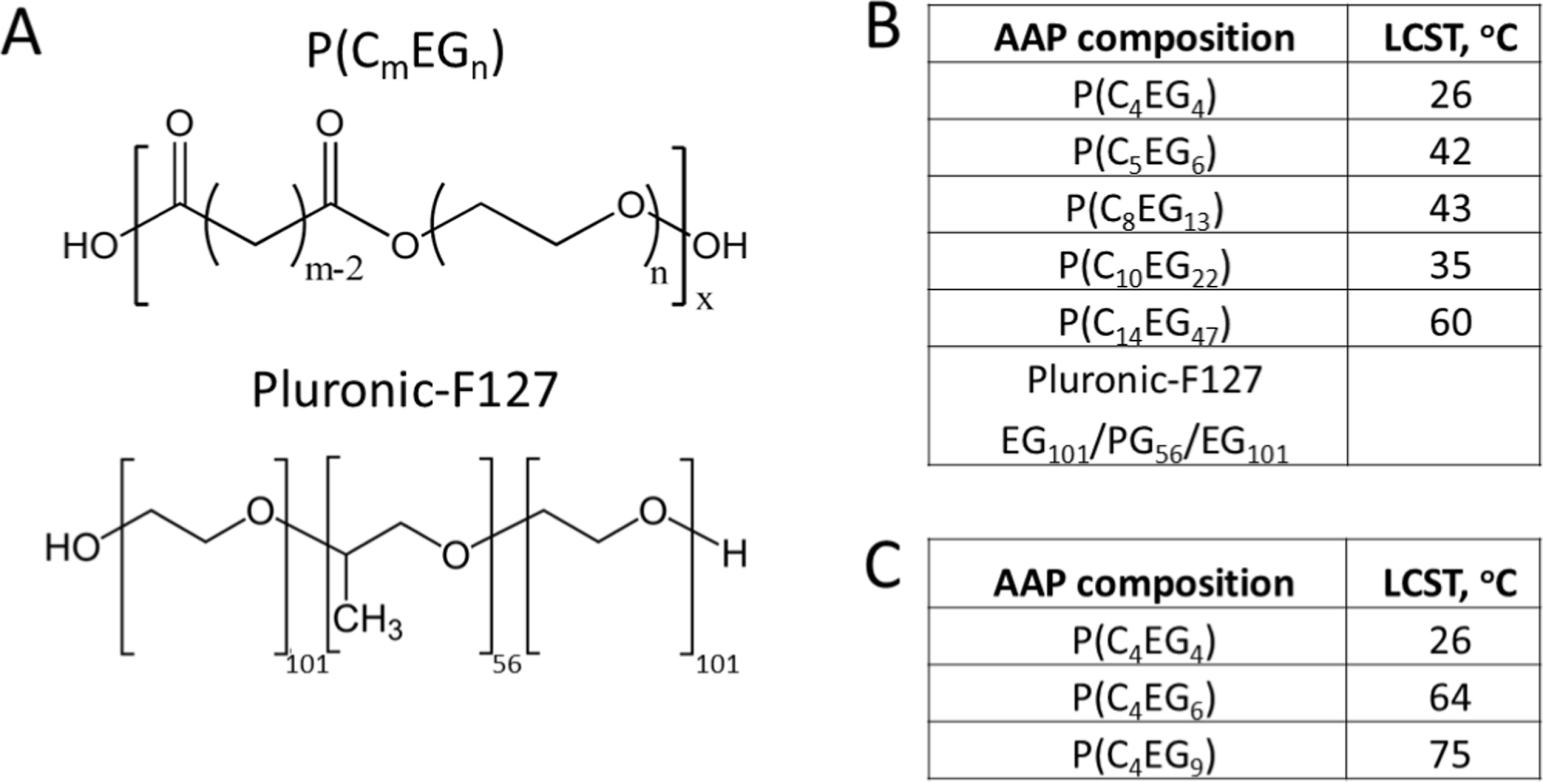
(A) Chemical Structure of P­(C_*m*_EG_*n*_) and Pluronic-F127; (B,C) Compositions and LCST Values of the Polymers Used for the Translocation Experiments

The samples varied the lengths of both the hydrophobic and the hydrophilic units (B) or the polymer polarity by keeping the length of the hydrophobic unit constant and increasing the hydrophilic size (C).

### Pulsed-Field Gradient NMR

#### Sample Preparation

Dry lipids were dispersed in D_2_O at the concentration of 20 mg/mL for 30 min above the lipid transition temperature, followed by 5 freeze/thaw cycles. Large unilamellar vesicles (LUVs) were prepared from this dispersion using an Avanti mini extruder. The vesicles were extruded 21 times through a 100 nm polycarbonate membrane and, afterward, 21 times through a 50 nm membrane, while the lipid was above its phase-transition temperature. AAPs were dissolved in D_2_O and added directly to the LUV solution before measurements.

#### Pulsed-Field Gradient NMR Measurements

The PFG NMR measurements were performed using a Varian 600 MHz system equipped with a diffusion ^1^H probe head. The attenuation of the spin echo signal from a pulse sequence containing a magnetic field gradient pulse was used to measure the translational diffusion of the molecules (hydrogens) in the sample at the time scales from ten to a few hundred milliseconds. During this time, hydrogens were able to overcome the distances of the order of hundreds of nanometers. Diffusion spin echo decays were measured using a standard stimulated echo (STE) pulsed-field gradient sequence^17^ with convection compensation. The measurement temperature was 25 °C if not mentioned explicitly. Observation times Δ were equal to 100 ms. The gradient pulse length δ was 2 ms. The kinetic PFG NMR measurements were performed at gradient pulse amplitude *G* chosen such that only the integrated polymer is observed. The number of scans per point was chosen depending on the kinetic rates, from 4 scans for the fastest processes to 128 scans for the slowest. The amplitude of the spin echo was determined as a function of time.

For all polymers and liposomes, the self-diffusion coefficients were measured in separate conventional PFG-NMR experiments on their aqueous solutions prior to translocation experiments. The relaxation times *T*_1_ and *T*_2_ have typical values of 1 and 0.5 s for all sample types, respectively, allowing conventional diffusion measurements at the time scale of the order of 0.5–1 s.

#### Fit Method of Kinetic PFG NMR Data

The PFG-NMR data were fitted using Bayesian inference using the Markov Chain Monte Carlo (MCMC) method as implemented in the Python package *emcee*.^18^ An uninformative prior was used. It allows us to look for a probability distribution of all possible solutions, which can be visualized using the Python module *corner*.*py*. The radius of LUVs and bilayer thicknesses used for the fits were 44 and 4 nm, respectively.

### Neutron Reflectometry (NR)

Neutron reflectometry (NR) is a powerful tool to determine the composition of thin films in a transverse orientation. A monochromatic collimated neutron beam of the wavelength λ hits the sample at a small angle θ, and the specularly reflected beam is measured as a function of momentum transfer *q*_*z*_ = (4π/λ) sin θ. Analysis of the reflectivity curve allows for recovering a scattering length density profile (SLD) of the layered sample in a transverse orientation. SLD is defined as ρ­(*z*) = ∑_*j*_ *n*_*j*_ *b*_*j*_, where *n*_*j*_ and *b*_*j*_ are the number density and scattering length of nuclei *j*, respectively, and contain full information on the structure and composition of each layer.

The SLD profile is constructed of multiple layers starting from the Si block with a thin SiO_2_ layer, a thin water layer, and a complex bilayer structure completed by the bulk water. The bilayer structure is built from two leaflets with each having a lipid headgroup and a lipid tail region, where the tail regions have contact. Penetration of the polymer in the inner or outer leaflet (tail plus respective headgroup region) results in a change of the respective scattering length density. An additional polymer brush layer is modeled as an additional layer of the polymer with solvent water penetration.

Neutron reflectivity data were acquired at the MARIA neutron reflectometer operated by Jülich Centre for Neutron Science at Heinz Maier-Leibnitz Zentrum in Garching (Germany).^19^ Custom temperature-regulated liquid cells were used to perform measurements of supported lipid bilayers.^20^ The measurements were performed using two different wavelengths, 10 Å for the low-*q* region and 5 Å for the high-*q* region up to 0.25 Å^–1^, with a 10% wavelength spread. The change of solvent contrast in the liquid cells was performed using a combination of valves and a peristaltic pump at small flow rates of ≈0.5 mL/min. The measurement temperature was 35 °C. All the fits were performed by the open-source Python-based software *anaklasis*.^21^ The used model is described in Supporting Information.

#### Preparation of the Supported Lipid Bilayer for NR

Two ultrapolished Si blocks (Siliciumbearbeitung Andrea Holm GmbH, Germany, rms roughness 1–2 Å, dimensions 150 × 50 × 20 mm) were cleaned with water and ethanol and treated with a UV–ozone plasma etching (Novascan technologies) for 10 min 2 times. Then, the liquid cells were assembled immediately, and H_2_O was injected into the cell. The liquid cell is made of boron glass and sealed by a Viton O-ring.^20^ After alignment of the sample with respect to the beam, the thickness and roughness of the silicon oxide layer were characterized. The liposomes were prepared in H_2_O similar to PFG NMR and had a concentration of 6 mg/mL. The vesicles were kept for 10 min at 35 °C to fuse on the substrate. Afterward, the remaining liposomes were flushed out with H_2_O. Now the samples were measured first in H_2_O and afterward in D_2_O to enable precise characterization of the lipid bilayer SLD profile by the simultaneous fit of the two contrasts. The polymers were solubilized in water at the desired concentration and manually injected into the cells with a syringe.

## Results and Discussion

We used AAPs of the P­(C_*m*_EG_*n*_) type, consisting of hydrophobic dicarboxylic acids (C_*m*_) and hydrophilic polyethylene glycol (EG_*n*_) oligomers (Scheme 1A). Here, *m* is the number of carbon atoms in the dicarboxylic acid backbone and *n* is the number of ethylene glycol monomers per hydrophilic unit of the AAP. AAPs with short hydrophobic and hydrophilic units solubilize in water as single chains when the hydrophilic unit dominates. With an increasing hydrophobic unit length, the length of the hydrophilic units must increase exponentially in order to keep water solubility. In the other case, micelle and gel formation takes place.^13^ For the translocation studies, we used only AAPs which solubilize in water as single chains.

To study the influence of AAP unit length on translocation through lipid membranes, we examined the translocation kinetics of seven AAPs with different unit lengths and, as a limiting case, the PEG_101_-PPG_56_-PEG_101_ triblock copolymer (Pluronic-F127, Scheme 1), having long hydrophilic and hydrophobic blocks. In this study, we varied both the lengths of the hydrophilic and the hydrophobic units simultaneously.

The translocation of AAPs through lipid membranes was studied by time evolution pulsed-field gradient (PFG) NMR, which allows measuring the kinetics of integrating the AAPs into the lipid vesicles. A schematic description of the experiment is presented in Figure 1. Large unilamellar vesicles (LUVs) of 44 nm radius made of 1-palmitoyl-2-oleoyl-*sn*-glycero-3-phosphocholine (POPC) are mixed with AAPs just before the measurement. Conventional PFG NMR, which allows measuring the mobility of every specific resonance nuclei (in our case hydrogen) in the time window of ∼10–1000 ms, easily distinguishes between the diffusion of free AAP and AAP integrated into the LUV. Time evolution PFG NMR allows one to measure how the number of AAPs integrated into LUVs changes with time by fixing the gradient at the value where the fast diffusion of free AAPs gets out of the measurement window.

**Figure 1:**
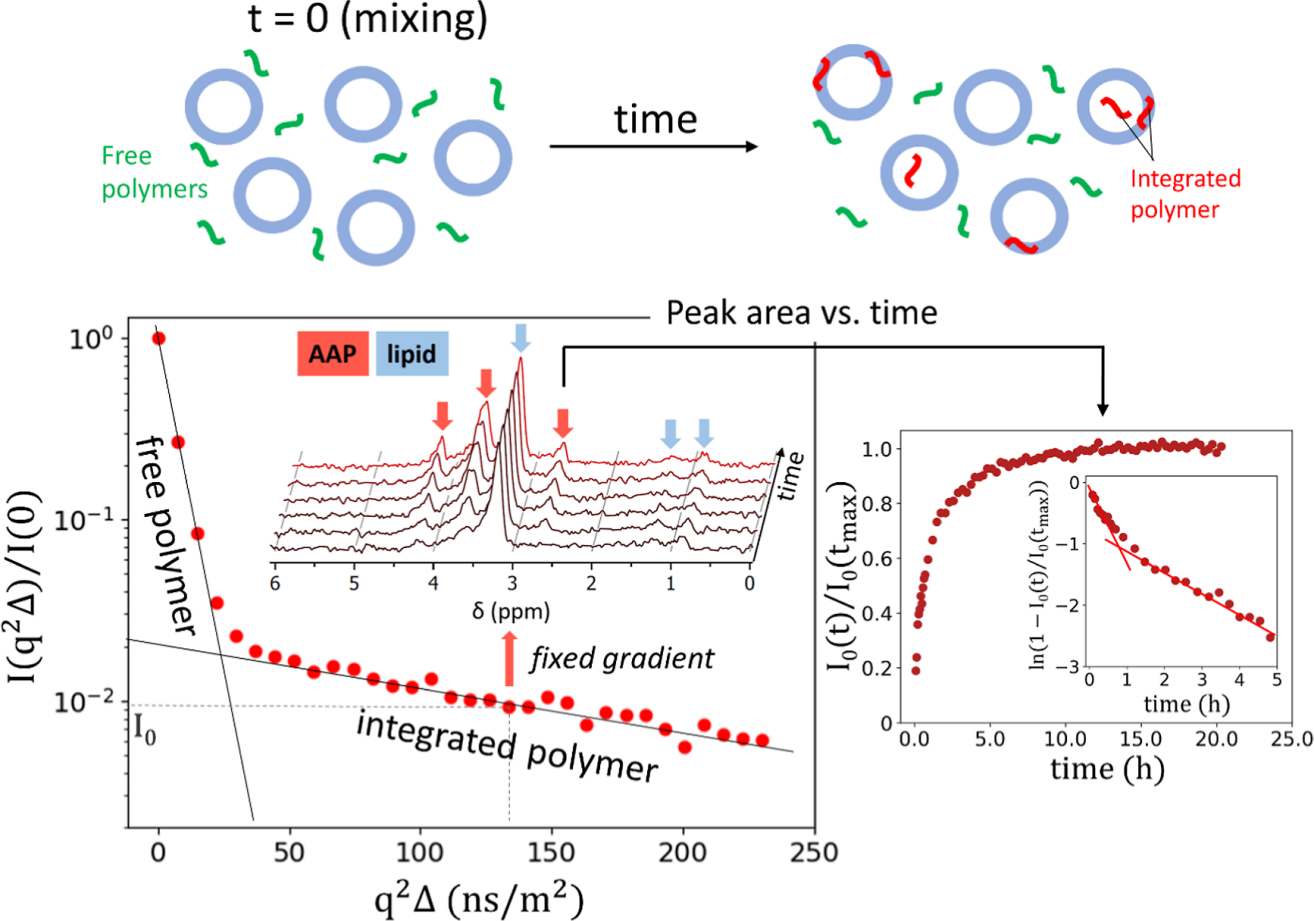
Schematic representation of the translocation experiment. The sketch above illustrates the time evolution of the LUV–AAP system and the difference between the free and LUV-integrated polymers. The plot below describes the principle of the time evolution PFG NMR technique. PFG NMR data present a Stokes–Einstein relation *I*(*q* ^2^)/*I*(0) ≈ exp­(−*q* ^2^Δ*D*) for a species with diffusion constant *D* visible as a linear region in a log plot. The wavenumber *q* is defined as *q* = γδ*g*, with γ as the gyromagnetic ratio for hydrogens, Δ = 20 ms as the diffusion time, and *g* as the magnetic field gradient. The examined EG_*n*_ signal at 3.6 ppm (red arrow) was used for the analysis of polymer diffusion at a fixed *q* ^2^Δ. The measured intensity *I*_0_(*t*) is normalized by *I*_*t*_(*t*_max_), where *t*_max_ is the time point where the kinetics reached saturation. An exemplary kinetic curve is shown on the right. The inset shows the same data as ln­(1 – *I*_0_ (*t*)/*I*_0_(*t*_max_)), indicating a two-step process as described in Supporting Information. Here, the initial slope (indicated as a line) is related to the adsorption rate *k*_a_ for nearly empty LUV, the slope at long times (indicated as a line) for nearly saturated LUV is related to the desorption rate *k*_d_ filling mainly the inside of the LUV, and the crossover intensity is related to the saturation concentration of the membrane. The same measurement also allows monitoring the time evolution of the lipid signals, which were shown to be stable during the measurement.

It was shown in our previous study that the resulting kinetic curves have two components related to adsorption of polymers onto the surface or into the membrane volume and desorption as the opposite effect, both occurring at both sides of the membrane.^9^ The minimal model describes the kinetics by a set of rate equations (Supporting Information) taking the limited LUV volume and limited AAP concentration in the membrane into account. The resulting two-step process is dominated by adsorption of the AAP to the membrane at shorter times and desorption to the inner LUV volume at longer times when the membrane is already saturated (see Figure 1 inset). Therefore, translocation parameters that are obtained from the fit are the adsorption rate (*k*_a_), the desorption rate (*k*_d_), and the concentration of AAP in the membrane (*c*_pm_).

### Influence of Hydrophobic/Hydrophilic Unit Length on Translocation

Figure 2A shows kinetic curves at 25 °C for three different polymers having similar molecular weights (MWs): P­(C_5_EG_6_), P­(C_10_EG_22_), and Pluronic-F127. Increasing the hydrophobic and hydrophilic unit lengths from P­(C_5_EG_6_) to P­(C_10_EG_22_) clearly slows the translocation process. Surprisingly, Pluronic-F127 also shows a translocation behavior at 25 °C, which is comparable to the AAPs. In another report, this process was detected only for higher temperatures.^12^ Figure 2 shows that the Pluronic-F127 translocation is qualitatively different with an extremely fast increase in the beginning due to a fast adsorption. Fitting of the kinetic curves with the model described above allows us to separate the adsorption kinetics and kinetics of filling the inner LUV volume, which is shown in Figure 2B. The adsorption is very rapid for all the investigated polymers, showing that the concentration of AAP in the membrane is constant during the major part of the translocation process. Increasing the hydrophobic and hydrophilic unit lengths from P­(C_5_EG_6_) to P­(C_10_EG_22_) increases the concentration of AAP in the membrane; however, these concentrations are much smaller than that of Pluronic-F127, explaining the difference at shorter times.

**Figure 2:**
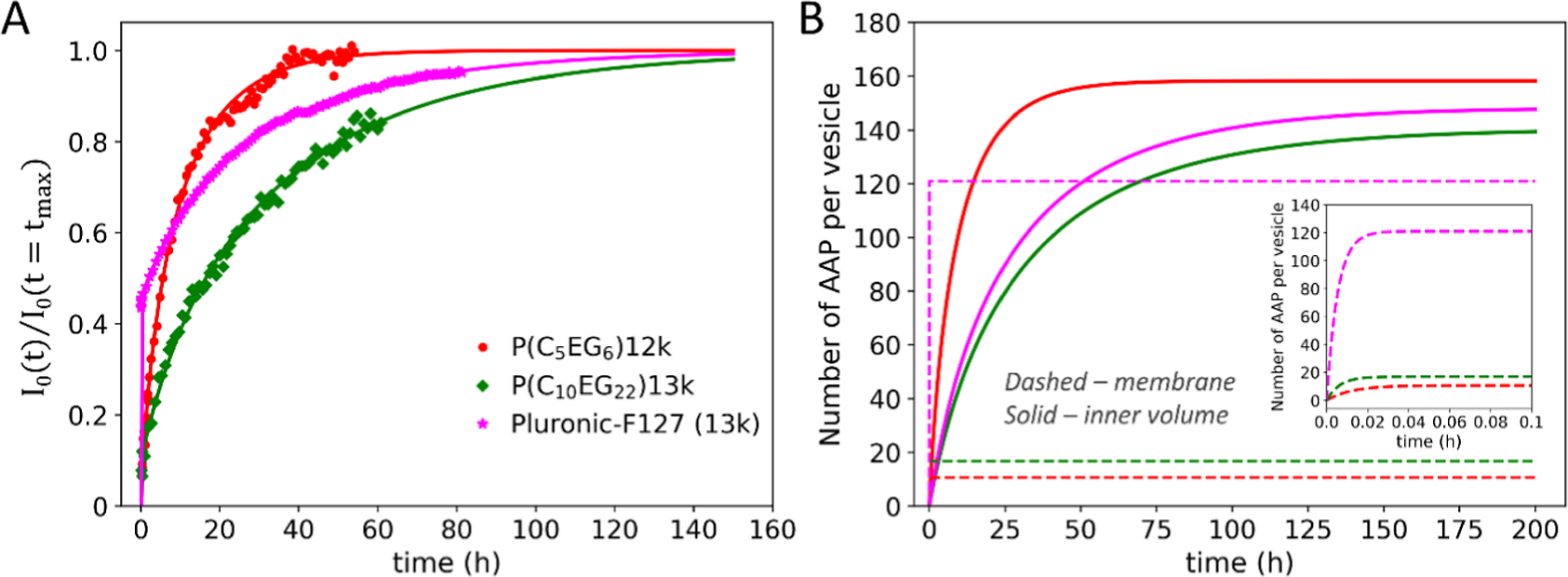
(A) Translocation kinetics for the AAPs P­(C_5_EG_6_)­12k, P­(C_10_EG_22_)­13k, and Pluronic-F127 (13k) were measured by time-resolved PFG NMR. (B) Kinetics of membrane and inner LUV volume saturation, reconstructed after the fit of kinetic curves from (A). The curves show the number of polymer molecules per vesicle in/at the membrane and in the inner volume. The color code is the same as in (A). The inset shows the same plot for membrane saturation zoomed in at shorter times. The membrane is saturated after a few minutes.

The MW-dependent rate constants *k*_a_ and partition coefficients between membrane and water solubilities (*c*_pm_/*c*_po_) are summarized in Figures 3 and 4 for the series P­(C_4_EG_4_), P­(C_5_EG_6_), P­(C_8_EG_13_), P­(C_10_EG_22_), P­(C_14_EG_47_), and Pluronic-F127. For P­(C_10_EG_22_) and P­(C_14_EG_47_), the PEG units already have molecular weights of 1 and 2 kg/mol, respectively, and as a consequence, the lowest MW samples already have overall MWs > 10 kg/mol for reasonably large numbers of repeat units. As larger-molecular-weight AAPs have too slow kinetics and were out of the measurement window of the PFG NMR method, the data for these AAP compositions are presented only for single MWs.

**Figure 3:**
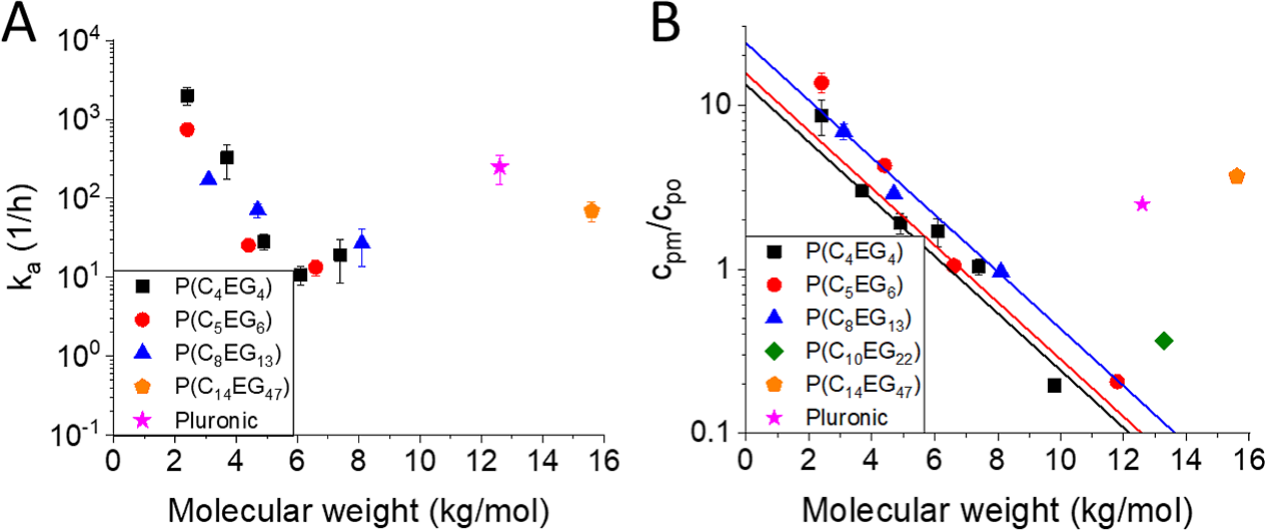
Dependence of *k*_a_ (A) and of polymer concentration in the membrane (B) on polymer molecular weight for different polymer compositions. For P­(C_10_EG_22_), the data quality does not allow us to obtain a reliable value of *k*_a_.

**Figure 4:**
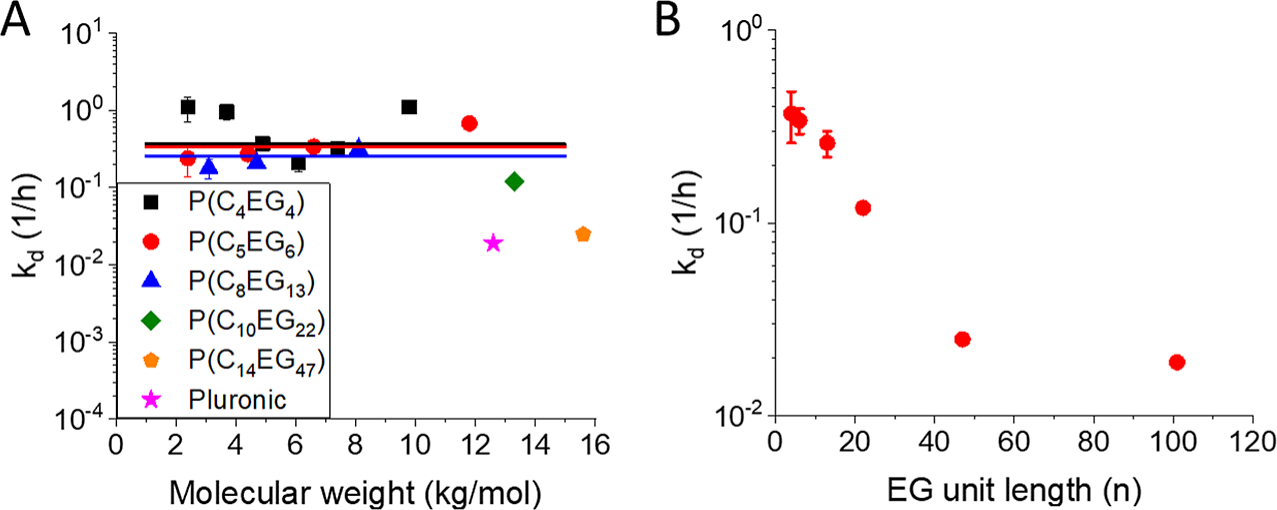
(A) Dependence of *k*_d_ on polymer molecular weight was observed for different polymer compositions. Solid lines: fits of the data assuming no MW dependence. (B) Dependence of the average *k*_d_ values obtained from (A) as a function of the hydrophilic unit length is given as the number of ethylene glycol monomers per hydrophilic unit of the AAP.

The adsorption rates *k*_a_ for the AAPs P­(C_4_EG_4_), P­(C_5_EG_6_), and P­(C_8_EG_13_) show a strong dependence on the polymer MW, but there is no significant difference from each other (Figure 3A). However, a trend of decreasing MW dependence from P­(C_4_EG_4_) to P­(C_8_EG_13_) can be observed. For these AAPs, as well as for P­(C_10_EG_22_), *k*_a_ cannot be determined for MWs larger than 8–9 kg/mol because of the relatively small concentration in the membrane and the small signal-to-noise ratio of the initial measurement period. Both P­(C_14_EG_47_)­16k and Pluronic-F127 show *k*_a_ values similar to those of the AAPs with shorter units and MWs of 3–5 kg/mol. The partitioning of the AAPs P­(C_4_EG_4_), P­(C_5_EG_6_), and P­(C_8_EG_13_) between the membrane and water also strongly depends on the MW (Figure 3B). For a polymer concentration in water of 1 wt %, the corresponding concentrations in the membrane vary in the range of 0.1–10 wt %. Fitting the dependences with the same slope gives intercepts that slightly increase from P­(C_4_EG_4_) to P­(C_8_EG_13_), indicating a larger interaction with the membrane for polymers equipped with longer units. P­(C_10_EG_22_), P­(C_14_EG_47_), and Pluronic-F127 follow this trend when compared with other AAPs having similar MW. The polymer concentration in the membrane not only depends on the polymer composition but also increases with temperature. This was tested for P­(C_4_EG_4_) as shown in Figure S2.

Figure 4A shows the desorption rate, *k*_d_ for polymers of different compositions. It was shown in our previous work that *k*_d_ shows almost no dependence on the AAP molecular weight for P­(C_4_EG_4_), and the large error in the *k*_d_ is due to a small signal-to-noise ratio of the initial data points.^9^ Therefore, for the polymers P­(C_4_EG_4_), P­(C_5_EG_6_), and P­(C_8_EG_13_), average error-weighted values of *k*_d_ were used for the comparison of different AAP compositions. The average *k*_d_ values of these polymers with smaller EG unit lengths already show a trend toward the reduction of *k*_d_ with increasing AAP unit lengths (Figure 4B). For P­(C_10_EG_22_), P­(C_14_EG_47_), and Pluronic-F127, *k*_d_ becomes increasingly smaller. We conclude that increasing the unit lengths leads to slower desorption. It is interesting to notice that the concentration in the membrane and *k*_d_ show opposite dependences with increasing length of the hydrophilic and hydrophobic units. Therefore, the effective translocation time, which can be defined as the time when the polymer concentration inside the vesicle reaches (1–1/*e*) of the concentration outside, does not decrease so dramatically with increasing the unit length.

In order to understand the dependence of the translocation process on the polymer unit length, we examined the polymer location in the membrane by neutron reflectometry. This method allows us to investigate the chemical composition of layered structures in a transverse orientation with Å precision. The information on the structure and composition of each layer is contained in the scattering length density (SLD) profile, which is modeled from the reflectivity curves. From this profile, the polymer location in the membrane can be detected if appropriate contrast conditions are chosen.

P­(C_4_EG_4_) and Pluronic-F127 were examined as extreme cases. The membrane was prepared by vesicle fusion on a Si/SiO_2_ substrate in D_2_O. High contrast between the polymer and membrane was obtained by using the fully deuterated lipid POPC-*d*_82_ with SLD close to D_2_O and hydrogenous polymers having strong contrast compared to the solvent D_2_O. The polymer in or at the membrane reduces the SLD of the lipid region or of the adjacent solvent significantly, dependent on its location. The reflectivity curves are fitted with a model that involves variable penetration of the AAP chains in the two leaflets and a potential external polymer layer above the membrane. The model is described in detail including tables with fit parameters in Materials and Methods and Supporting Information.

In order to increase polymer concentration in the membrane, these measurements were performed at 35 °C. The experiments at different concentrations show that the P­(C_4_EG_4_)­2k chains are fully solubilized in the hydrophobic interior of the membrane (Figure 5). The preferred localization of the AAP chains in the outer leaflet of the membrane is attributed to the less fluid nature of the inner membrane due to its proximity to the supporting substrate and was seen before for the solubilization of other molecules in lipid membranes.^22^ The mass concentrations of polymer in the tail region calculated from the SLD profile are 3.8% and 21.6% for polymer concentrations in the aqueous phase of 1% and 5%, respectively. The partition value obtained from the PFG NMR is 9.6% for 1% concentration when extrapolated to 35 °C (see Figure S2), which is close to the concentration of 7.5% in the outer leaflet obtained from NR. The value could not be directly measured by PFG NMR due to very fast translocation at 35 °C, which is beyond the time resolution of the technique.

**Figure 5:**
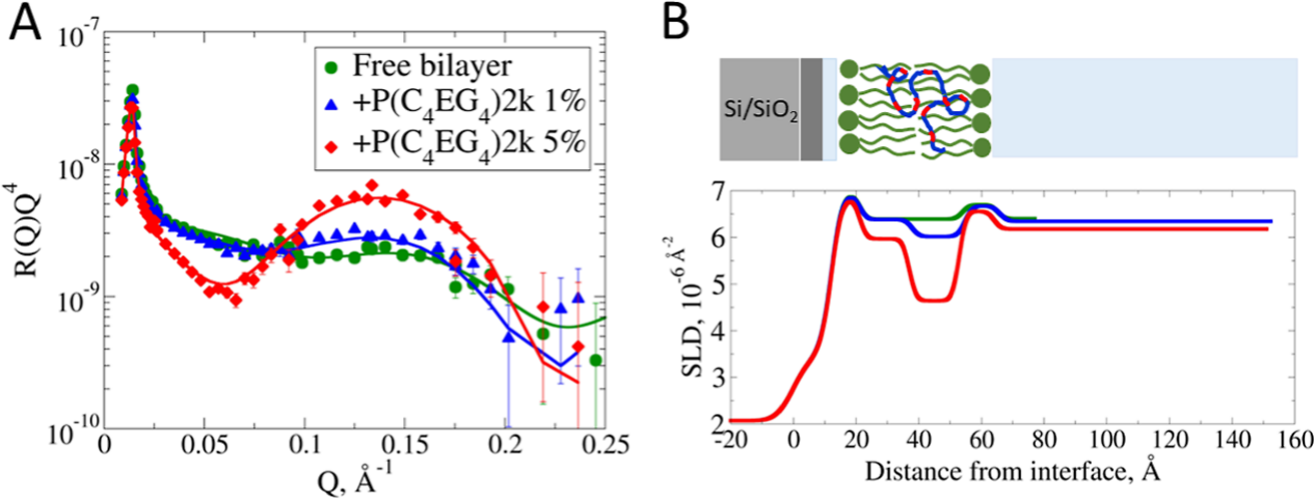
(A) Neutron reflectivity curves in RQ^4^ representation (symbols) and fits (lines) for a fully deuterated POPC lipid bilayer in D_2_O (green) and after incubation with P­(C_4_EG_4_)­2k of 1 and 5 wt % concentration (blue and red, respectively). Solid lines represent the fits with a solvent penetration model. (B) Scattering length density profiles and the corresponding solvent penetration model (see Materials and Methods) were obtained from the fit. We observe that the AAP is mostly localized in the tail region of the membrane, preferably in the outer leaflet.

In contrast to the above, Pluronic-F127 behaves differently. The pronounced decrease in the SLD profile at the membrane interior and the flatter and more elongated decrease on the membrane surface (see Figure 6B, red arrows) are a result of the polymer incorporation. Due to the solubility of the hydrophobic PPG middle block in nonpolar solvents, we assume its location is unique in the membrane interior. The hydrophilic outer PEG blocks are located only at the membrane surface due to the incompatibility of longer PEG blocks with the membrane interior (Figure 6). This scenario is also in agreement with earlier theoretical work.^23^ The PPG mass concentration in the membrane calculated from the SLD profile is 9.4%, which is significantly larger than the 2.5% obtained from the PFG NMR study. We explain this by a strong decrease of the Pluronic-F127 critical micellar concentration (CMC) with increasing temperature,^24^ which also increases the interaction with lipid membranes.^11^

**Figure 6:**
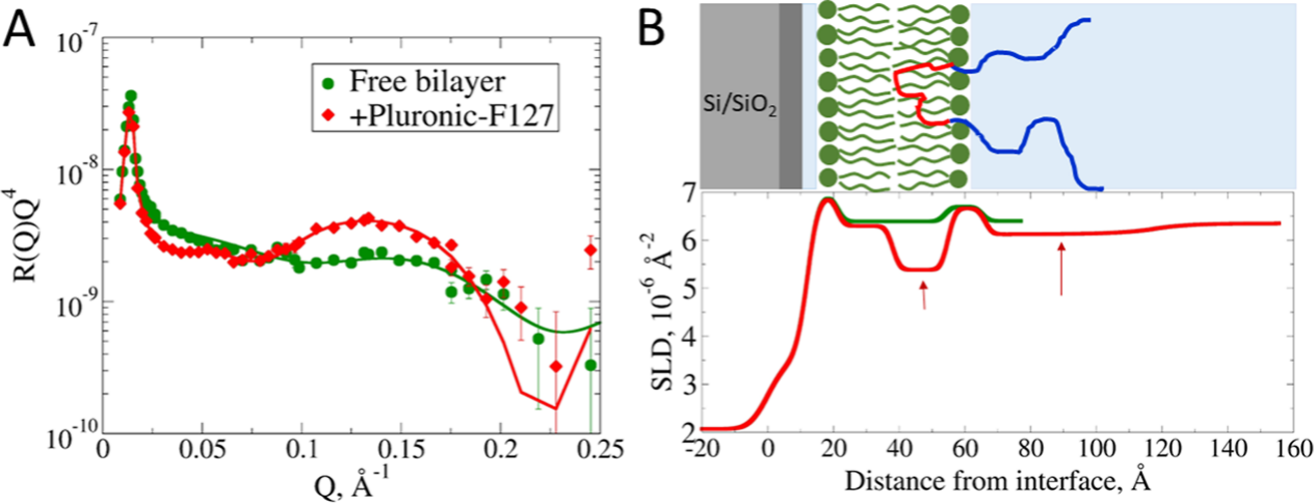
(A) Neutron reflectivity curves in RQ^4^ representation (symbols) and fits (lines) for a fully deuterated POPC lipid bilayer in D_2_O (green) and after incubation with EO/PO triblock (Pluronic-F127) of 1 wt % concentration (red). Solid lines represent the fits with a solvent penetration model. (B) Scattering length density profiles and corresponding solvent penetration model (see Materials and Methods) obtained from the fit. We observe a 36 Å extended layer with a reduced SLD compared to that of bulk water at the outer surface corresponding to the PEG outer blocks of Pluronic-F127.

### Influence of the Overall AAP Polarity on Translocation

Simulation studies predict the maximum rate for passive translocation to occur at an intermediate polarity level, where the polymer polarity is balanced both with the hydrophobic membrane interior and with the aqueous medium. On both sides from this point, the translocation rate is predicted to decrease dramatically.^14,15^ However, applications might require more hydrophilic membrane translocating polymers to maintain good water solubility at higher temperatures. In order to investigate the influence of polymer polarity on translocation, we studied the series of P­(C_4_EG_4_), P­(C_4_EG_6_), and P­(C_4_EG_9_), where the overall polarity continuously increases, and accordingly, the LCST values increase from 26 to 75 °C (Scheme 1B). Very short diacid units in combination with relatively short PEG units yield AAPs with a relatively homogeneous polarity profile. According to the results from the previous section (Figures 3 and 4), block length is not expected to play a major role for this set of AAPs; therefore, all the significant differences between them must be attributed to the AAP polarity.

The dependences of MW on the translocation behavior of these AAPs are shown in Figure 7. The decrease of *k*_a_ with increasing polymer LCST results from the increase of the free energy of adsorption Δ*G*_a_ (Figure 7C). The polymers having higher LCSTs are better soluble in water and less soluble in the membrane interior. Therefore, the interaction parameter with water is smaller and with the membrane interior is larger, which leads to an increase of the adsorption enthalpy and consequently of the free energy. With increasing LCST, the polymer entropy is not expected to change a lot when the temperature is well below LCST, so the main effect comes from enthalpy changes.

**Figure 7:**
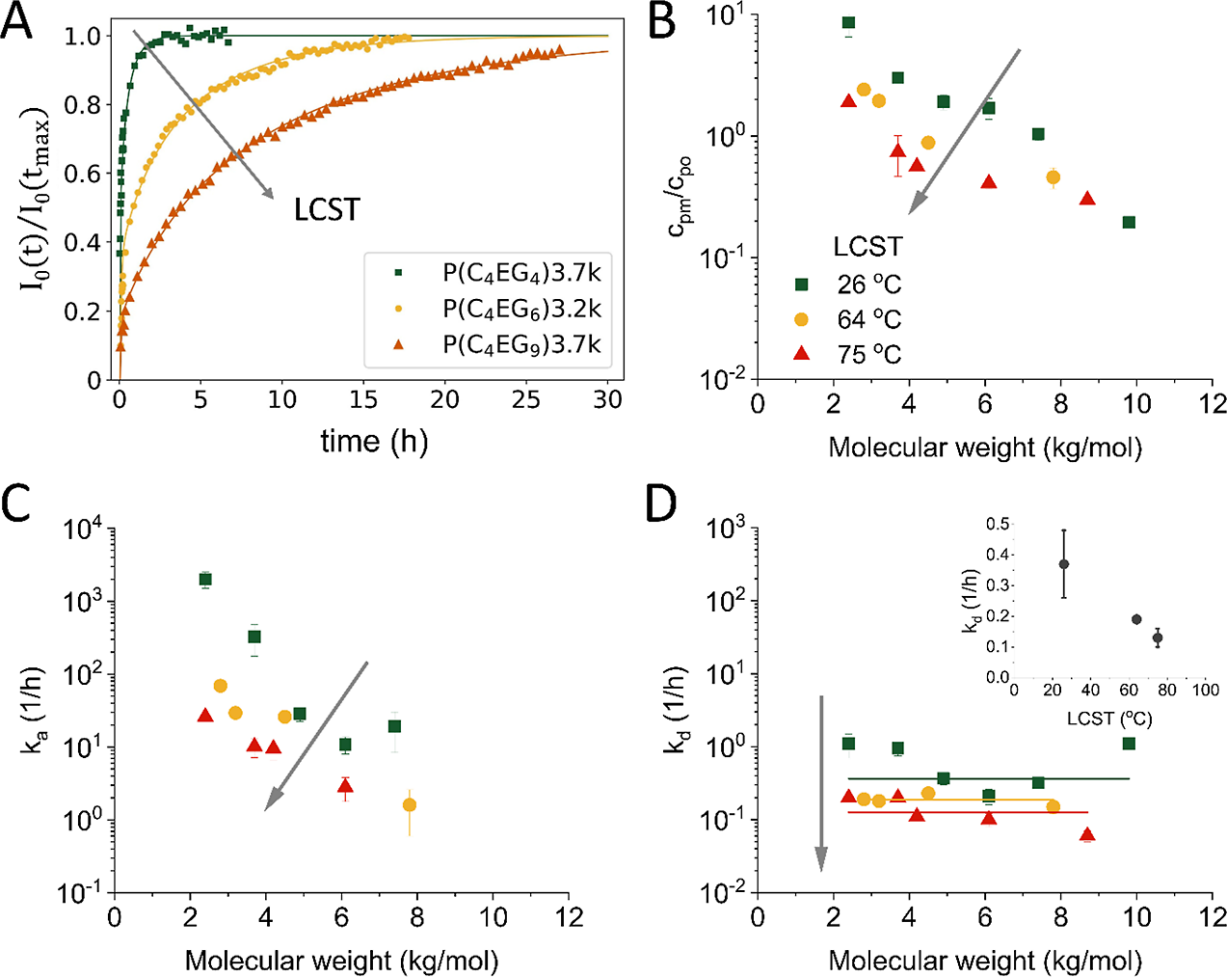
Translocation behavior through POPC membranes at *T* = 25 °C of P­(C_4_EG_4_), P­(C_4_EG_6_), and P­(C_4_EG_9_) having LCST values of 26, 64, and 75 °C, respectively. (A) Kinetic curves for AAPs had similar MW. (B) Dependence of the partition coefficient *c*_pm_/*c*_po_ on polymer MW. (C) Dependence of the adsorption rate *k*_a_ on the polymer MW. (D) Dependence of the desorption rate *k*_d_ on polymer MW. The inset shows the dependence of the average *k*_d_ on polymer LCST. The gray arrow in all the plots shows the direction of LCST increase.

The desorption rate *k*_d_ shows a weak decrease with increasing LCST (Figure 7D), which is slightly more expressed than in the series of P­(C_4_EG_4_)–P­(C_8_EG_13_), where there is also a smaller LCST increase from P­(C_4_EG_4_) to P­(C_8_EG_13_).

The change from P­(C_4_EG_4_) (LCST = 26 °C) to P­(C_4_EG_9_) (LCST = 75 °C) causes lower membrane solubility and slower adsorption and desorption processes. As a result, the translocation process is slower by a factor of 10. However, the most hydrophilic polymer still shows visible translocation and can be used to transport hydrophobic molecules through the membrane.

### Translocation Mechanisms of Polymers with Different Amphiphilicities and Polarities

AAPs containing small hydrophilic and hydrophobic units have an almost homogeneous polarity profile along the polymer chain and a low amphiphilic character. Due to the incorporation of the diacid units, these polymers are less polar than PEG homopolymers and soluble in both water and hydrophobic environments. The PFG-NMR results show for P­(C_4_EG_4_) a strong incorporation within the lipid membrane. The neutron reflectivity results (Figure 5) document the solubilization of the polymer chains in the hydrophobic membrane interior. We conclude that the AAP polymer chains translocate through the lipid membrane as a whole entity, as shown in Figure 8A.

**Figure 8:**
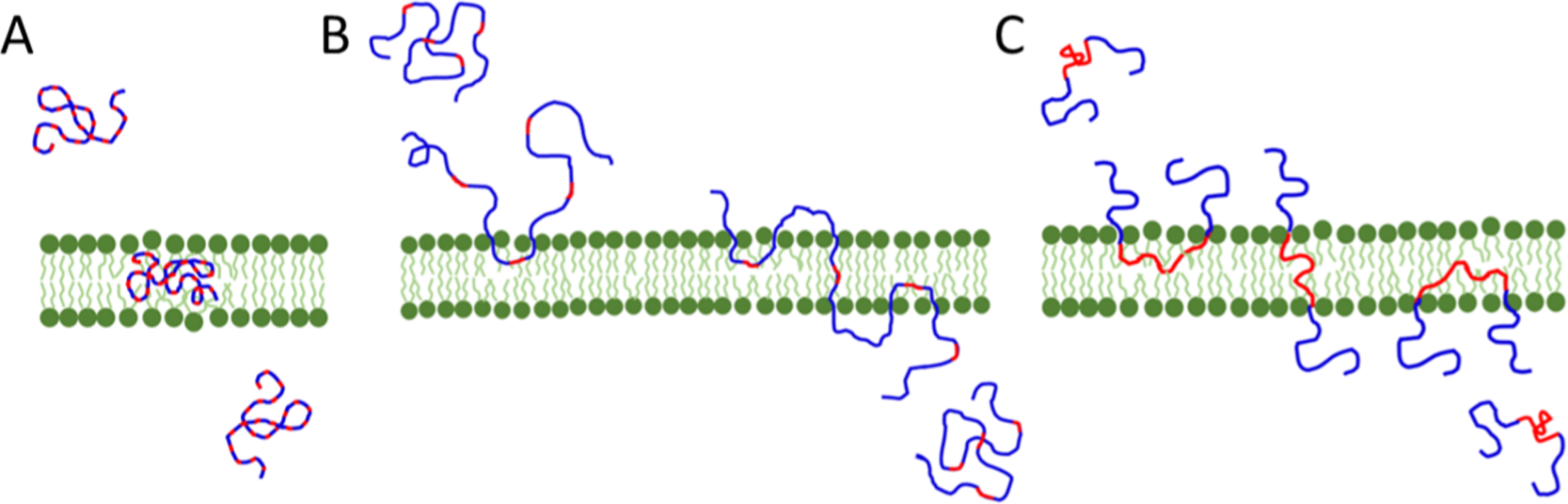
Schematic illustration of translocation mechanisms of AAPs having a more homogeneous polarity profile (A), amphiphilic AAPs (B), and the Pluronic triblock (C).

In the series P­(C_4_EG_4_), P­(C_4_EG_6_), and P­(C_4_EG_9_), the overall polarity increases, but the polarity profile can still be considered as largely homogeneous due to the short hydrophilic units. The polarity increase causes especially a reduction of *k*_a_ and *c*_pm_ (Figure 7). For this reason, the translocation process becomes slower with increasing polarity.

The scenario is different if both hydrophilic and hydrophobic units are increased in size. In order to keep the AAPs water-soluble, a linear increase of the hydrophobic unit must be compensated by an exponential increase of the PEG unit.^13^ Such dependence rises from the contribution of these units to the free energy of micellization, where the enthalpic term depends linearly on the hydrophobic unit length and the entropic term depends logarithmically on the total polymer length, mainly the PEG length. AAPs P­(C_5_EG_6_) and P­(C_8_EG_13_) still fit into the scenario described for P­(C_4_EG_4_). Interestingly, there is a small trend toward higher membrane solubility (Figure 3B), although the volume fraction of the hydrophilic component increases. The trend toward an increasing membrane concentration continues for P­(C_10_EG_22_), P­(C_14_EG_47_), and the Pluronic triblock. These polymers have PEG unit molecular weights of 1, 2, and 4 kg/mol, respectively. PEGs of such sizes are insoluble in nonpolar solvents, and therefore, the solubilization of these polymers inside the bilayer is not expected. This assumption is confirmed by the neutron reflectivity examination of the Pluronic triblock. The scattering results show that the hydrophobic PPG middle block is anchored in the membrane with the PEG blocks being solubilized in the aqueous phase.

The membrane decoration of the Pluronic triblock is accompanied by translocation. The required hopping of the PEG chains to the opposite membrane side (Figure 8C) most likely is facilitated by the incorporated PPG blocks, which make the membrane locally more polar and increase the level of disordering of the lipid tails. The lipid disordering by incorporation of Pluronic-F127 also dramatically accelerates the flip-flop process of lipid molecules,^25^ which is rather slow (>1 h^–1^) in pure membranes.^26^

As the triblocks are anchored only with the hydrophobic blocks in the membrane, their membrane concentration can be higher compared to that of the AAPs, which are fully solubilized in the membrane. The same scenario can explain the high membrane concentration of P­(C_14_EG_47_). In comparison with P­(C_4_EG_4_), the increased lengths of the hydrophilic and hydrophobic units lead to an amphiphilic polymer with a distinct polarity profile. The long PEG units are no longer compatible with the membrane interior, so the polymer is adsorbed to the membrane only via the hydrophobic units. On the other hand, for AAPs with longer hydrophilic units, loop formation is entropically less disfavored. This makes configurations where EG units are outside of the hydrophobic membrane interior more favorable and allows a higher polymer density at the membrane, as observed in the PFG-NMR study (Figure 3B). We assume that the translocation mechanism of this AAP is similar to Pluronic-F127, with the difference that in this case, several hydrophobic units are initially adsorbed to the membrane. Again, the translocation happens via the hopping of the hydrophilic units to the opposite membrane side (Figure 8B). Although the number of hopping steps per AAP chain needs to be larger, the smaller EG units compared to the ones of the Pluronic triblock make the process faster.

The high *k*_a_ values of the amphiphilic Pluronic triblock and P­(C_14_EG_47_) polymers (Figure 3A) agree with an adsorption process, where only the hydrophobic units are solubilized in the membrane. On the other hand, for the polymers with short hydrophilic and hydrophobic units, the adsorption step comprises the slower solubilization of the whole chain in the membrane. For the release process, the scenario is opposite. Due to the slow hopping process of the long PEG units, *k*_d_ is slowed for the Pluronic triblock and P­(C_14_EG_47_) (Figure 4). The other polymers in this series, in particular P­(C_8_EG_13_) and P­(C_10_EG_22_), lie in-between the extreme cases of P­(C_4_EG_4_) and P­(C_14_EG_47_).

In order to compare the overall translocation behavior of different polymer types with regard to an application as a drug carrier, the filling kinetics of the inner vesicle volume are shown in Figure 9 for P­(C_4_EG_4_), P­(C_4_EG_6_), P­(C_14_EG_47_), and Pluronic-F127. Due to the strong MW dependence of the translocation process, the AAPs of a similar molecular weight of 16 kg/mol were compared. For P­(C_4_EG_6_), the MW of 16 kg/mol was not measured due to the very long required measurement time; therefore, its filling kinetics was modeled based on the *k*_a_, *k*_d_, and *c*_pm_ values extrapolated to the 16k, assuming a simple exponential MW dependence of *k*_a_ and *c*_pm_ and a MW-independent *k*_d_ value. For this reason, this curve is equipped with a larger error bar. Interestingly, P­(C_4_EG_4_) and P­(C_14_EG_47_) behave almost similarly. At first sight, this is surprising as P­(C_4_EG_4_) shows a medium polarity and is compatible with both the aqueous environment and the membrane interior. P­(C_14_EG_47_) is not expected to be an ideal candidate for translocation because of the long PEG units. The slow hopping process of these units to the opposite membrane side, however, is compensated by the high concentration of polymer adsorbed to the membrane. The same scenario applies to a lesser extent to the Pluronic triblock. From the direct comparison with the higher MW P­(C_14_EG_47_), it seems that the AAP chain architecture is advantageous for the translocation process. It is also interesting that P­(C_4_EG_6_) shows a very slow translocation behavior, although its composition is not drastically different from that of P­(C_4_EG_4_), and the average polarity based on the LCST behavior is similar to that of P­(C_14_EG_47_). But the combination of a lowered membrane concentration and low *k*_a_ and *k*_d_ values results in a slow translocation process.

**Figure 9:**
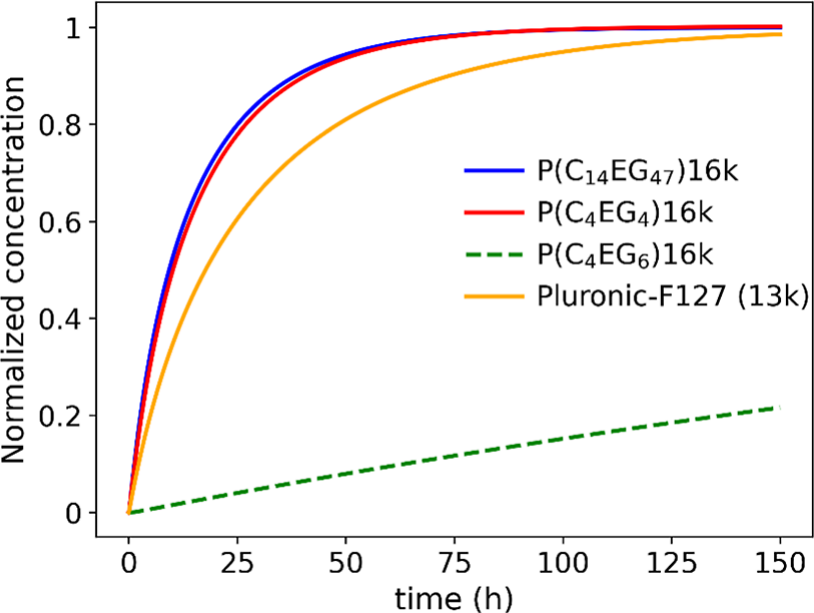
Filling kinetics of the inner vesicle volume. The curves for P­(C_4_EG_4_)­16k, P­(C_14_EG_47_)­16k, and Pluronic-F127 are obtained from fitting of the respective PFG NMR data; the curve for P­(C_4_EG_6_)­16k is calculated from the extrapolation of *k*_a_, *k*_d_, and *c*_pm_ to 16 kg/mol.

These findings correlate with the simulation results on random amphiphilic copolymers.^15^ Random copolymers where the maximum unit length is fixed to a small value behave similarly to homopolymers. They solubilize in the membrane and translocate. On the opposite, random copolymers containing longer hydrophobic and hydrophilic units better adsorb to the membrane due to the ability of loop formation at the membrane surface but have a smaller translocation rate. The decrease in the adsorption and translocation with increasing polymer hydrophilicity in our study also goes along with the simulation results, although it is difficult to compare the hydrophobicity values in the simulation with the experimental scenario.

## Conclusions

Our results show that the translocation of polymer molecules through lipid membranes happens for a very wide compositional range. For AAPs, both the hydrophilic and hydrophobic unit lengths as well as the overall hydrophilicity influence the translocation properties. The combination of short hydrophilic PEG and hydrophobic dicarboxylic acid units yields polymers with a low amphiphilic character. If the hydrophobic unit is not too dominant, the polymers still solubilize in water and in the hydrophobic environment of the membrane interior. As a result, these polymers translocate through lipid membranes. Keeping the amphiphilic character on a low level and increasing the polarity drastically slows the translocation kinetics due to reduced compatibility with the membrane interior. The increase of both the hydrophilic and hydrophobic unit lengths leads to amphiphilic polymers. In order to keep them water-soluble, long PEG units are required, and the AAP solubilizes only with the hydrophobic units in the membrane. This allows relatively high AAP concentrations at the membrane and, as a consequence, a fast translocation process. Triblock copolymers can behave similarly. However, the cmc of Pluronic-F127, used in this work, is relatively high. According to literature reports, it is about 1 g/L.^27,28^ Other block copolymers equipped with more hydrophobic blocks than PPO show significantly lower CMCs, and it is likely that the interaction with the membrane is so strong that it acts as a trap for the polymer and thus prevents translocation. Apart from the translocation speed, the use of more hydrophilic AAP for the translocation process is advantageous as these polymers are equipped with LCST values well above the body temperature and have the potential to carry hydrophobic loads such as drug molecules without solubility difficulties. AAPs are especially useful for such applications as their interfacial activity and emulsification properties are low due to the alternating architecture.

## Supplementary Material



## Notes

Polymer synthesis, kinetic model for fitting the PFG NMR data, fits of the neutron reflectometry data, and temperature dependence of polymer concentration in the membrane (PDF)
