# Supplementary material for: Exploring the Limits of Passive Macromolecular Translocation through Phospholipid Membranes
Source: Biomacromolecules. 2025 Sep 12;26(10):6917–26. doi: 10.1021/acs.biomac.5c01234 (PMC12522124; doi:10.1021/acs.biomac.5c01234)
Supplement: Supplementary file 1 [file bm5c01234_si_001.pdf]

SUPPORTING INFORMATION

Exploring the limits of passive macromolecular translocation through phospholipid membranes

*Ekaterina Kostyurina[1], Ralf Biehl[1], Margarita Kruteva[1], Alexandros Koutsioubas[2], Henrich Frielinghaus[2], Nageshwar Rao Yepuri[3], Stephan Förster[1], Jürgen Allgaier[1]**

[1]Jülich Centre for Neutron Science (JCNS-1), Forschungszentrum Jülich GmbH, Leo Brandt Straße, 52425 Jülich, Germany

[2]Jülich Centre for Neutron Science (JCNS), Heinz Maier-Leibnitz Zentrum (MLZ), Lichtenbergstrasse 1, 85748 Garching, Germany

[3]Australian Nuclear Science and Technology Organisation, National Deuteration Facility, New Illawarra Rd, Lucas Heights, New South Wales, Australia

Corresponding author: * j.allgaier@fz-juelich.de

Table of Contents

Polymer synthesis 2

Kinetic model for fitting the PFG NMR data 6

Fits of the Neutron Reflectivity data..7

Temperature dependence of polymer concentration in the membrane 10

References 10

Polymer synthesis

Synthesis and fractionation of P(C_4EG_4), P(C_5EG_6), P(C_8EG_{13}), P($C_{10}EG_{22}$) and P($C_{14}EG_{47}$) is described in a previous publication.[1] Synthesis and fractionation of P(C_4EG_6) and P(C_4EG_9) is described below.

Materials. Succinic anhydride (Sigma-Aldrich, ≥99.0%, batch purity 100.0% (GC)), was used as received. PEG 300 (Sigma-Aldrich; M_n = 298) and PEG 400 (Sigma-Aldrich; M_n = 404) were dried prior to use under high vacuum conditions overnight. The PEG molecular weights were determined by ^{1}H-NMR as described in the previous publication.[1] *p*-Toluenesulfonic acid monohydrate (Sigma-Aldrich, ≥99.0%) was used as received.

Synthesis of P(C_4EG_6)-R. 16.805 g of succinic anhydride (0.168 mol), 49.970 g of PEG 300 (M_n = 298, 0.168 mol) and 53 mg of *p*-toluenesulfonic acid were mixed and heated under argon overnight at 120 °C. Then, the pressure was decreased to 100 mbar and within 8 h the temperature was raised to 160 °C. The pressure was then reduced to 20 mbar (overnight), 6 mbar

(7h) and 10^{-2} mbar (3 days), still keeping the temperature at 160 °C. The reaction product $P(C_4EG_6)$-R was characterized by SEC/LS (Table S1).

Fractionation of $P(C_4EG_6)$-R. After removal of the solvent toluene, 48.8 g of the polymer was mixed with 1.8 L of dry ethanol and heated. At 64 °C the mixture became clear and was cooled to 60 °C. The phase separation occurred overnight and the lower phase ($P(C_4EG_6)$-FA) was isolated (2.2 g after drying). It was mixed with 400 mL of THF and 670 mL of dry ethanol. Upon heating to 34 °C the mixture became clear. It was cooled to 22 °C overnight and the lower phase formed was isolated ($P(C_4EG_6)$15k) (1.1 g after drying). Then the upper pase of the ethanol mixture of $P(C_4EG_6)$-R was cooled from 60 °C to 55 °C. The lower phase formed ($P(C_4EG_6)$8k) was separated (4.1 g after removal of solvent) and the new upper phase was cooled to 40 °C. Then again the lower phase ($P(C_4EG_6)$7k was separated (12.1 g after removal of solvent) and the upper phase was cooled to 35 °C. The lower phase formed ($P(C_4EG_6)$5k) was separated (1.5 g after removal of solvent) and the new upper phase was cooled to 26 °C. The precipitate was isolated ($P(C_4EG_6)$4k, 4.6 g after removal of solvent). Further cooling to 10 °C and -10 °C yielded the samples $P(C_4EG_6)$3k (7.0 g) and $P(C_4EG_6)$3ka (4.0 g), respectively. The SEC/LS characterization results are summarized in Table S1.

Table S1. SEC/LS characterization results of $P(C_4EG_4)$ polymers.

Sample name	M_n, g/mol	M_w/M_n
$P(C_4EG_6)$-R	4,950	1.34
$P(C_4EG_6)$15k	15,200	1.14
$P(C_4EG_6)$8k	7,800	1.21
$P(C_4EG_6)$7k	6,500	1.11
$P(C_4EG_6)$5k	4,500	1.16
$P(C_4EG_6)$4k	4,400	1.09
$P(C_4EG_6)$3k	3,200	1.11
$P(C_4EG_6)$3ka	2,800	1.10

Synthesis of $P(C_4EG_9)$-R. 12.396 g of succinic anhydride (0.124 mol), 50.068 g of PEG 400 (M_n = 404, 0.124 mol) and 290 mg of *p*-toluenesulfonic acid were dissolved in 140 mL of toluene. The mixture was heated to the boiling point in a flask equipped with a Dean-Stark apparatus. Within 16 h 2.0 mL of water was extracted. Then the Dean-Stark apparatus was exchanged by a modified Dean-Stark apparatus, containing molecular sieve 0.4 nm as a drying agent (see Figure S1), and the water extraction was continued for 6 hours. The reaction product $P(C_4EG_9)$-R was characterized by SEC/LS (Table S2).

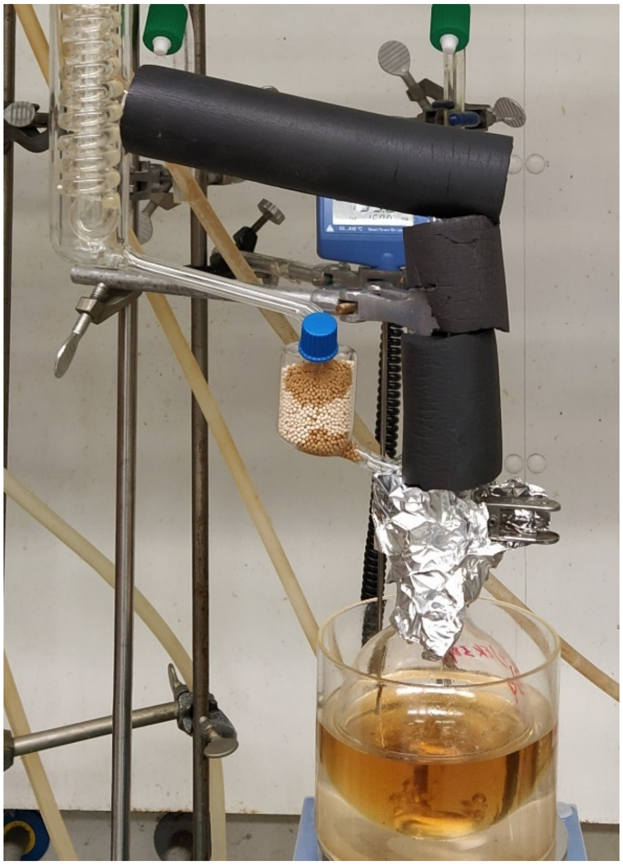

Figure S1. Modified Dean-Stark apparatus containing a molecular sieve as a drying agent. The upper glass tubing is covered for insulation purposes.

Fractionation of $P(C_4EG_9)$-R. After removal of most of the solvent toluene, the polymer was mixed with 2.0 L of dry ethanol and heated. At 59 °C the mixture became clear and was slowly cooled to 35 °C. The phase separation occurred overnight and the lower phase ($P(C_4EG_9)$-FA) was isolated. The uper phase was mixed with 100 mL of dry ethanol, warmed until the mixture became clear and then cooled to 25 °C overnight. The lower phase formed ($P(C_4EG_9)$6k) was

separated (4.3 g after removal of solvent) and the new upper phase was cooled to 10 °C. The lower phase (P(C_4EG_9)4k was separated (6.5 g after removal of solvent) and the upper phase was mixed with 100 mL of dry ethanol and cooled to -10 °C. Again, the lower phase (P(C_4EG_9)3k was separated (3.9 g after removal of solvent)) and the upper phase was now cooled to -22 °C. The solid precipitate was isolated (P(C_4EG_9)2k, 5.2 g after removal of solvent).

In the next step, P(C_4EG_9)-FA was mixed with 1.0 L of THF and 2.6 L fo dry ethanol. The mixture became clear at 32 °C and was cooled to 24 °C overnight. The lower phase (P(C_4EG_9)16k was separated (6.9 g after removal of solvent)) and the upper phase was mixed with 1.0 L of dry ethanol. It was heated, became clear at 30 °C and was cooled overnight to 23 °C. The lower phase was isolated (P(C_4EG_9)12k, 6.5 g after removal of solvent). The upper phase was cooled to 8 °C overnight. The lower phase formed was isolated (P(C_4EG_9)9k, 8.0 g after removal of solvent). The upper phase was further cooled to – 10 °C and the precipitate isolated the next day (P(C_4EG_9)7k, 3.7 g after removal of solvent). The SEC/LS characterization results are summarized in Table S2.

Table S2. SEC/LS characterization results of P(C_4EG_4) polymers.

Sample name	M_n, g/mol	M_w/M_n
P(C_4EG_9)-R	6,070	1.65
P(C_4EG_9)16k	16,200	1.16
P(C_4EG_9)12k	12,300	1.09
P(C_4EG_9)9k	8,700	1.09
P(C_4EG_9)7k	6,700	1.05
P(C_4EG_9)6k	5,900	1.13
P(C_4EG_9)4k	4,200	1.11
P(C_4EG_9)3k	3,600	1.08
P(C_4EG_9)2k	2,400	1.09

Kinetic model for fitting the PFG NMR data

The following set of rate equations is used to describe changes in the total number of AAP molecules in the outside volume (po), in or adsorbed to the membrane (pm), and inside the vesicles (pi):[2]

$$\frac{dn_{po}}{V_o dt} = k_d \cdot \frac{n_{pm}}{V_m} - k_a \cdot c_{po} \cdot \left(1 - \frac{n_{pm}}{n_{pmax}}\right) \quad (1)$$

$$\frac{dn_{pm}}{V_m dt} = k_a \cdot c_{po} \cdot \left(1 - \frac{n_{pm}}{n_{pmax}}\right) - 2 \cdot k_d \cdot \frac{n_{pm}}{V_m} + k_a \cdot \frac{n_{pi}}{V_i}\left(1 - \frac{n_{pm}}{n_{pmax}}\right) \quad (2)$$

$$\frac{dn_{pi}}{V_i dt} = k_d \cdot \frac{n_{pm}}{V_m} - k_a \cdot \frac{n_{pi}}{V_i}\left(1 - \frac{n_{pm}}{n_{pmax}}\right) \quad (3)$$

where k_a and k_d are the translocation rates for the adsorption and desorption processes respectively, n_j and c_j indicate the respective numbers and number concentration of AAP molecules in the respective volumes V_j. $c_{pmax} = \frac{n_{pmax}}{V_m}$ is the maximum number concentration in the membrane. Because of the small total liposome volume of about 4%, the final equilibrium c_{po} was regarded to be constant over the whole measurement time. The system of rate equations can be solved numerically (boundary condition t=0: $c_{pi} = 0; c_{pm} = 0, c_{po} = const$) and is normalized to the final equilibrium integrated number $n_{pm} + n_{pi}$. We note that a simpler system of rate equations, e.g. two equations neglecting the membrane volume, cannot reproduce the observed two step behavior. To describe the measured data, we fit k_a, k_d and n_{pmax} by Bayesian analysis also to retrieve statistical errors. The equilibrium value of n_{pm} is recovered by modeling the kinetic curves with the fitted values without normalization. It should be noted that accessing the LUV-integrated AAP amount together with the limited Vi allows to discriminate between adsorption, desorption and c_{pm}, which cannot be achieved by other methods.

Fits of the Neutron Reflectivity data

Before adding the polymers, supported lipid bilayers (SLB) were characterized in two contrasts (using H_2O and D_2O as a solvent). A summary of the simultaneous bilayer fit is presented in Table S3. Bilayers in the presence of polymers were measured at one contrast (D_2O) therefore the parameters were set more restricted. Their summaries are presented in Table S4, S5 and S6 for P(C4EG4)2k 1% concentration, P(C4EG4)2k 5% concentration and Pluronic respectively.

Table S3. Parameters used in the fit model. Cells marked with green color represent free parameters which were fitted, and the parameters marked by the same symbols (*) or (**) were bound to stay the same. Roughness of the Si/SiO_2 substrate and thickness of SiO_2 was determined in the preliminary measurement. Values for the lipid SLD were used from the literature.[3]

**Layer**	**Thickness, Å**	**SLD, 10^{-6} Å^{-2}**	**Solvent penetration, %**	**Roughness, Å**
Si	-	2.07	0	4.34
SiO_2	11.8	3.47	0	3
Water (H_2O/D_2O)	0.74 ± 0.22	-0.54 ± 0.02 / 6.40 ± 0.001 (*)	100	1.75 ± 0.59 (**)
Inner head groups	8.61 ± 0.37	7.35	43.5 ± 2.7	1.75 ± 0.59 (**)
Tail region	32.8 ± 0.54	6.39	2.4 ± 0.4	1.75 ± 0.59 (**)
Outer head groups	11.2 ± 0.51	7.35	68.6 ± 1.2	1.75 ± 0.59 (**)
Bulk water (H_2O/D_2O)	-	-0.54 ± 0.02 / 6.40 ± 0.001 (*)	100	-

Table S4. Parameters used in the fit model for the sample with P(C_4EG_4)2k of 1% concentration. Cells marked with green color represent free parameters which were fitted, and the parameters marked by the same symbols (*) or (**) were bound to stay the same. Values which were fixed during the fit were taken from the free bilayer fit (Table S3). Additional layer after the outer head groups was added to determine a possible localisation of polymer chain at the outer bilayer surface. 100% solvent penetration let us conclude that there is no detectable amount of polymer at the surface.

Layer	Thickness, Å	SLD, 10^{-6} Å^{-2}	Solvent penetration, %	Roughness, Å
Si	-	2.07	0	4.34
SiO_2	11.8	3.47	0	3
Water (H_2O/D_2O)	0.74	6.36 ± 0.01 (*)	100	1.75
Inner head groups	8.61	7.35	43.5	1.75
Inner tail region	16.6 ± 0.36 (**)	6.39 ± 0.007	2.4	1.75
Outer tail region	16.6 ± 0.36 (**)	5.98 ± 0.02	2.4	1.75
Outer head groups	11.2	7.35	68.6	1.75
Polymer brush	60.4 ± 55.6	0.69	100 ± 0.2	43.2 ± 11.7
Bulk water (H_2O/D_2O)	-	6.36 ± 0.01 (*)	100	-

Table S5. Parameters used in the fit model for the sample with P(C_4EG_4)2k of 5% concentration. Cells marked with green color represent free parameters which were fitted, and the parameters marked by the same symbols (*) or (**) were bound to stay the same. Values which were fixed during the fit were taken from the free bilayer fit (Table S3). Additional layer after the outer head groups was added to determine a possible localisation of polymer chain at the outer bilayer surface. More than 99% solvent penetration together with unreasonably large value of thickness let us conclude that there is no detectable amount of polymer at the suface.

Layer	Thickness, Å	SLD, 10^{-6} Å^{-2}	Solvent penetration, %	Roughness, Å
Si	-	2.07	0	4.34
SiO_2	11.8	3.47	0	3
Water (H_2O/D_2O)	0.74	6.20 ± 0.01 (*)	100	1.75
Inner head groups	8.61	7.35	43.5	1.75
Inner tail region	15.9 ± 0.14 (**)	5.94 ± 0.03	2.4	1.75
Outer tail region	15.9 ± 0.14 (**)	4.60 ± 0.02	2.4	1.75
Outer head groups	11.2	7.35	68.6	1.75
Polymer brush	199 ± 25.5	0.69	99.1 ± 0.2	12.6 ± 13.1
Bulk water (H_2O/D_2O)	-	6.20 ± 0.01 (*)	100	-

Table S6. Parameters used in the fit model for the sample with Pluronic-F127 1% concentration. Cells marked with green color represent free parameters which were fitted, and the parameters marked by the same symbols (*) or (**) were bound to stay the same. Values which were fixed

during the fit were taken from the free bilayer fit (Table S3). Additional layer after the outer head groups was added to determine a possible localisation of polymer chain at the outer bilayer surface. Fit shows that there is a layer of polymer brush at the bilayer surface.

**Layer**	**Thickness, Å**	**SLD, 10^{-6} Å^{-2}**	**Solvent penetration, %**	**Roughness, Å**
Si	-	2.07	0	4.34
SiO_2	11.8	3.47	0	3
Water (H_2O/D_2O)	0.74	6.35 ± 0.02 (*)	100	1.75
Inner head groups	8.61	7.35	43.5	1.75
Inner tail region	16.6 ± 0.18 (**)	6.31 ± 0.02	2.4	1.75
Outer tail region	16.6 ± 0.18 (**)	5.34 ± 0.02	2.4	1.75
Outer head groups	11.2	7.35	68.6	1.75
Polymer brush	50.6 ± 3.1	0.69	96.0 ± 0.4	12.9 ± 5.9
Bulk water (H_2O/D_2O)	-	6.35 ± 0.02 (*)	100	-

Temperature dependence of polymer concentration in the membrane

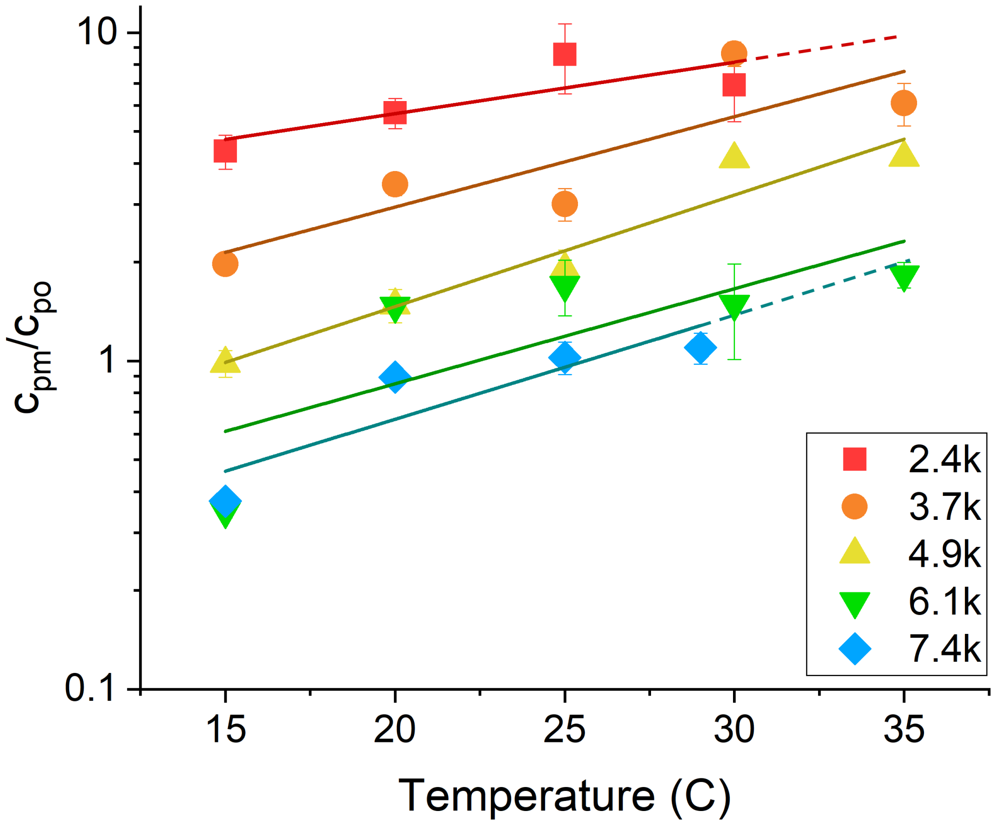

Figure S1. Temperature dependence of polymer concentration in the membrane for AAP $P(C_4EG_4)$ of different MW. Solid lines show linear fits in the log scaled figure. The dashed lines are the extrapolations of the fitted lines to 35°C. The extrapolated value for the polymer $P(C_4EG_4)$2.4k has been used in the comparison with the NR data and equals 9.6.

References

[1] E. Kostyurina, J. U. De Mel, A. Vasilyeva, M. Kruteva, H. Frielinghaus, M. Dulle, L. Barnsley, S. Förster, G. J. Schneider, R. Biehl, J. Allgaier, *Macromolecules* **2022**, *55*, 1552–1565.

[2] E. Kostyurina, J. Allgaier, M. Kruteva, H. Frielinghaus, A. Csiszár, S. Förster, R. Biehl, *J. Am. Chem. Soc.* **2022**, *2022*, 15348–15354.

[3] N. R. Yepuri, T. A. Darwish, A. M. Krause-Heuer, A. E. Leung, R. Delhom, H. P. Wacklin, P. J. Holden, *Chempluschem* **2016**, *81*, 315–321.
